# Towards Practical Application of Paper based Printed Circuits: Capillarity Effectively Enhances Conductivity of the Thermoplastic Electrically Conductive Adhesives

**DOI:** 10.1038/srep06275

**Published:** 2014-09-03

**Authors:** Haoyi Wu, Sum Wai Chiang, Wei Lin, Cheng Yang, Zhuo Li, Jingping Liu, Xiaoya Cui, Feiyu Kang, Ching Ping Wong

**Affiliations:** 1 Division of Energy and Environments, Graduate School at Shenzhen, Tsinghua University, Xili University Town, Shenzhen City, China; 2 School of Materials Science and Engineering, Georgia Institute of Technology, 771 Ferst Drive, Atlanta, GA, 30332, USA; 3 Department of Electronic Engineering, Chinese University of Hong Kong, Shatin, NT, Hong Kong SAR, China; 4 Current address: IBM Albany Nanotechnology, 257 Fuller Road, Albany, NY, 12203.

## Abstract

Direct printing nanoparticle-based conductive inks onto paper substrates has encountered difficulties e.g. the nanoparticles are prone to penetrate into the pores of the paper and become partially segmented, and the necessary low-temperature-sintering process is harmful to the dimension-stability of paper. Here we prototyped the paper-based circuit substrate in combination with printed thermoplastic electrically conductive adhesives (ECA), which takes the advantage of the capillarity of paper and thus both the conductivity and mechanical robustness of the printed circuitsweredrastically improved without sintering process. For instance, the electrical resistivity of the ECA specimen on a pulp paper (6 × 10^−5^Ω·cm, with 50 wt% loading of Ag) was only 14% of that on PET film than that on PET film. This improvement has been found directly related to the sizing degree of paper, in agreement with the effective medium approximation simulation results in this work. The thermoplastic nature also enables excellent mechanical strength of the printed ECA to resist repeated folding. Considering the generality of the process and the wide acceptance of ECA technique in the modern electronic packages, this method may find vast applications in e.g. circuit boards, capacitive touch pads, and radio frequency identification antennas, which have been prototyped in the manuscript.

Paper based electronics have been drawing intensive attentions because of their low cost and environmental footprint^1^,^2^,^3^,^4^. Compared with the conventional printed circuit board (PCB) processes, e.g. etching the plated metal foil (subtractive method) and solder-reflowing at a high temperature for mounting the electronic components, paper substrates are more suitable for the dry and additive process because of their intrinsic characteristics of high moisture absorption and relatively low thermal stability. A few methods have been demonstrated to form highly conductive tracks on paper, such as metal sputtering^5^,^6^, direct-writing of conductive inks^7^,^8^, printing of liquid metal^9^ and inkjet-printing of conductive inks (metal nanoparticles)^10^,^11^,^12^,^13^,^14^,^15^ etc. All these methods have their own advantages. However, in order to realize practical applications, scientists and engineers are aiming at the development of new materials and techniques which do not involve high temperature, complicated process, and toxic and rare elements (e.g. indium). To be noted, even though conductive inks have been investigated for years, there still lack reports regarding a systemic evaluation of reliability, probably because the partially sintered nanoparticles of the printed conductive ink fine lines are prone to be oxidized. Moreover, the conductive nano-particles are prone to penetrate into the pores of ordinary paper and become partially segmented, which is disadvantageous to build up stable connectivity^16^.

One path toward practical and broad application of the paper-based electronics is to incorporate electrically conductive adhesives (ECAs) as the printed conductive matter for the paper based printed circuits. ECAs, a typical composite material, which is composed of micron-sized metal fillers and a polymer resin binder, have been used in the modern electronic packaging industry for decades for the purpose of electrical and mechanical interconnections^17^. The metal fillers are electrically conductive particles such as silver, copper or gold. Since silver shows the highest electrical conductivity, excellent adhesion to the substrate and highly chemical stability, it is applied as the ECA fillers in most cases. ECAs with silver fillers possess comparable and sometimes even better stability, reliability, conductivity and mechanical robustness, even compared with solder junctions and copper lines on conventional PCBs^18^,^19^. These features render them more practical and cost effective as compared with the nano-metal based conductive inks and many other novel materials. Years ago, Hick et al. reported that some ECA types printed onto hard card paper can render a drop of about 60% in electrical resistivity as compared with the same samples printed on smooth polymer films after being cured at some 120°C^20^. Yet further exploration of the feasibility of ECA circuits for paper-based electronics has not been explored. Possible reasons are: 1) most commercial ECAs are thermosetting ones which require a thermal curing step at temperatures higher than 150°C, which is not conducive to paper (even though amine-based curing agent can cure with epoxy resin at ambient temperature, the electrical conductivity of the ECA gets very poor); 2) without a curing process, thermoplastic ECAs usually have relatively high electrical resistivity (10^−4^Ω·cm and higher), which is one to two orders of magnitude higher than that of copper wiring and the commercial high performance PCBs and the commercial high performance thermosetting ECAs. In the present study, we customize a thermoplastic ECA for the use as printing circuit materials on paper.

Herein, a thermoplastic copolymer poly(butylmethacrylate-co-methyl methacrylate) (pBMA-co-MMA) is used as the resin binder for the ECA. This copolymer is widely used due to the excellent mechanical strength and flexibility. Moreover, it can be processed at mild temperatures, which is important for maintaining stable dimensions of paper substrates as compared with those using the thermosetting resin binders, which are necessarily involved with thermal curing steps. The ECA possesses excellent printability when dispensed in ordinary solvents, and it forms a decent conductive network on various paper substrates. For example, printed lines with 0.2 mm width and 0.4 mm pitch were obtained via screen printing the ECAs onto an ordinary printing paper, which showed comparable resolution with ordinary flexible printed circuits(see Fig. S1)^21^. Considering the fact that the thickness of most of the papers are higher than 100 μm, and the screen-printed ECA has the thickness much thinner than that, thus in this manuscript we didn't take into account the influence of the paper thickness. Future works which involve with the ultrathin paper shall consider the thickness issue. Like the commercial polymer-based additives commonly used in the paper-making industry, the polymer resin of the ECA partially infiltrates into the paper due to the capillary force from the paper substrate. This phenomenon enhances the Ohmic conductance of the silver microflake fillers that remain on top of paper (as shown in Fig. 1a). For example, with 50 wt% of Ag loading, a nearly 700% enhancement of electrical conductivity was achieved on paper than on a conventional PET film. We note that the roughness of paper may render the variation of conductivity about 20 ~ 30% since the surface roughness of pulp paper and filter paper is around 5 μm as comparing to the thickness of the printed ECA (~20 μm). Yet the obvious enhancement of conductivity (e.g. ~700% improvement) suggests that the roughness of paper only play a minor role in the altered electrical conductivity, as compared with the characteristic of absorptivity of paper substrates. The present approach of using the thermoplastic ECA to print circuits on the paper substrate not only relaxes the thermal budget for paper PCB technology but also improves the electrical conductivity; therefore, enabling a new integration scheme of paper PCBs into the current surface mount technology (SMT) to realize the “chip-on-a-flex” function for future green electronics with very low costs. It is also important to note that we focus on sustainable paper sources such as craft paper, newspaper, printing paper, filter paper and pure pulp paper, etc. The present work may provide insights into the design of novel paper based PCBs with eco-friendliness and low cost.

## Results and Discussion

**Figure 1:**
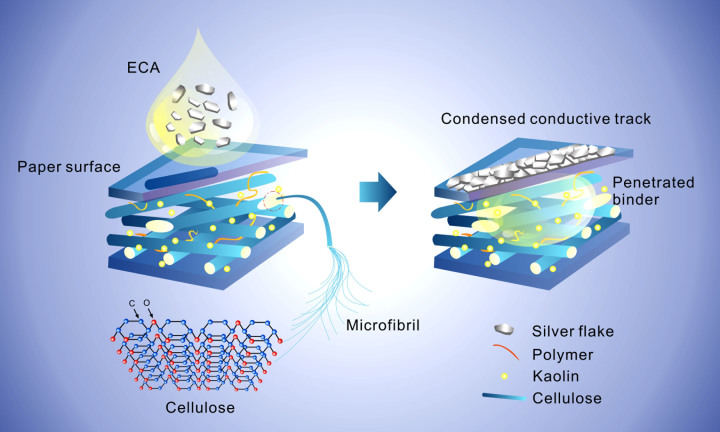
A schematic illustration of capillary force-assisted formation of condensed conductive track of ECAs. When the ECA is coated on the paper surface, the resin binder penetrates inside the paper, resulting in the increase of local silver loading at the surface.

**Figure 2:**
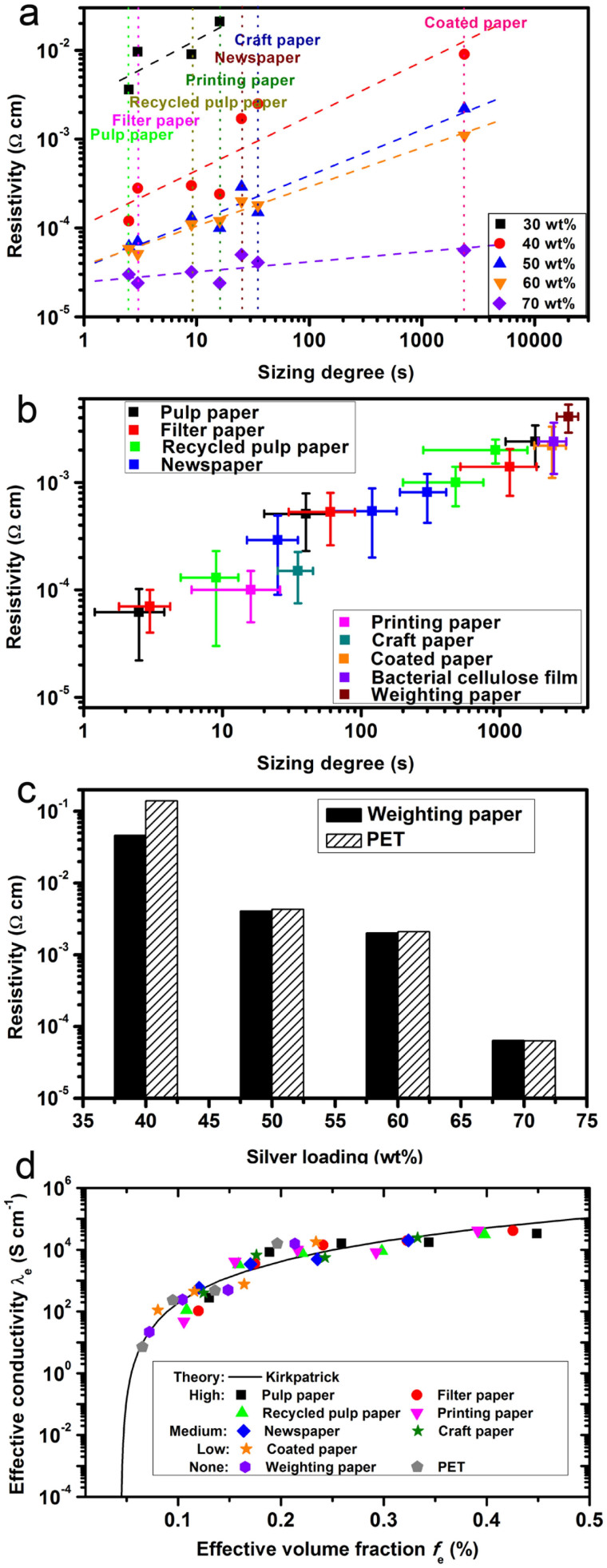
(a) The resistivity of the ECAs on various papers vs. the sizing degree of papers. (b) The resistivity of the ECA with 50 w% of silver fillers vs. the sizing degree of papers. (c) The resistivity of the ECA samples vs. the silver loading on both weighing paper and PET film. (d)The effective conductivity of the printed ECA vs. the effective volume fraction *f*_e_ for tested paper substrates. The simulated curve is based on a modified Kirkpatrick EMA model from equation (1), in which *f*_c_ = 0.045 and D = 3.0.

For microelectronic applications, the resistivity of the ECA lower than 1 × 10^−4^ Ω·cm is necessary, so as to avoid severe resistive loss^22^. Generally, when the ECA with micron-sized silver flake fillers is coated onto a surface, they tend to distribute randomly in the resin. In comparison, when ECA is applied to the paper surfaces, the resin tends to penetrate into the paper due to the capillary force^23^,^24^. As shown in Fig. 1, the porous nature of paper enables the absorption ability of the resin binder of ECA, which renders the silver fillers condense at the printed surface. This condensation process is valuable to the enhancement of electrical conductance and the soaked resin binder is more strongly bonded to the paper, when compared to the conventional smooth plastic substrates, e.g. PET (polyethylene terephthalate). As shown in Fig. 2c, with the silver content of 50 wt%, our tailor-made thermoplastic ECA showed electrical resistivity of 4.3 × 10^−3^ Ω·cm when printed on PET, which is almost the same to the ECA sample printed on a nonporous weighing paper, as shown in Fig. 2b. As compared, with the same silver filler loading level, the resistivity of the ECA on pulp paper showed about 6 × 10^−5^ Ω·cm, which is only 14% of that on PET film.

Considering there are composition, roughness, pore size and capillarity differences for various kinds of papers, it is impossible to explore the available papers using all above indexes. Moreover, the pore size of the papers may vary during different soaking situation, which renders significant uncertainty for experimental measurement. Fortunately, even though there are various factors, capillarity is the key factor because all other issues may render the change of capillarity, which can significantly influence the improvement of the electrical conductivity of the printed ECA. Besides, there is an index in the paper-making industry which clearly indicates the level of capillarity of the paper, i.e. the sizing degree. People use sizing degree to evaluate the level of capillarity; which is an index of absorption time of a typical ink (usually it is a standard matter) absorbed by a piece of paper^23^. The faster the ink is absorbed, the stronger the capillarity is and the weaker the sizing effect is. Inspired by this quantification method, we use the resin binder absorption time to represent the capillarity of various papers, and this absorption time can be described as the sizing degree toward this resin binder. In order to modulate the water-absorptivity and mechanical property, sizing agents (such as polyacrylamide and polyvinyl alcohol) are added to reduce capillarity^25^. In this way, we describe the order of various kinds of papers for the absorption of the binder of the ECA. Among our selected papers, pulp paper, filter paper, recycled pulp paper and printing paper represent the substrates with high capillarity. The newspaper and craft paper are typically the medium capillary substrates, and the coated paper and weighing paper are the substrates that have negligible capillarity. For comparison, since PET does not have capillary effect, in another word, its binder absorption time (sizing degree) is the highest (or infinity). We note that the recently reported interesting studies of transparent nano-paper has much inhibited pore structure, and thus they are not studied in this manuscript^26^.

Fig. 2a shows the electrical resistivity of ECA with various silver contents on the paper samples in more details. When ECA contains 30 wt% of silver fillers, only pulp paper, filter paper, recycled pulp paper and printing paper were conductive (lower than 10^6^Ω·cm), which showed the resistivity at the magnitude of 10^−3^ Ω·cm. When the silver content was increased to 40 wt%, the ECAs were conductive on all kinds of papers and the resistivity decreased with the increase of capillarity of substrates. A similar trend was observed for those ECAs with 50, 60 and 70 wt% of silver content. The variation of resistivity becomes smaller with the increase of silver loading in the ECA, probably due to the improved viscosity (Fig. 2a). Detailed relationship between the resistivity of ECA and the sizing degree of the papers is illustrated by printing the ECA (50 wt% silver loading) on the selected paper specimens (Fig. 2b). Herein, we employed the pulp paper, filter paper, recycled pulp paper and newspaper with medium and strong binder absorption ability (we tentatively coated the samples by a certain amount of polyvinyl alcohol and bacterial cellulose fibers correspondingly to adjust the sizing degree) for analysis. It clearly shows a trend that a lower resistivity is strongly correlated to a paper substrate with a shorter liquid-penetration time, which is determined by the capillarity of papers. ECA samples on those papers without adding sizing agents (e.g. pulp paper, filter paper and recycled pulp paper) showed the lowest resistivity, which was around 6.2 × 10^−5^ ~ 1.0 × 10^−4^ Ω·cm. After adding a proper amount of sizing agent, the absorption ability of the paper was inhibited and the resistivity of the coated ECA was increased by 2 ~ 10 folds. The resistivity was further increased when ECA was printed on the papers with a strong sizing degree. For example, after sizing the pulp paper, filter paper and recycled pulp paper, the ECA printed on them showed an increased resistivity of around 1.4 ~ 2.4 × 10^−3^ Ω·cm, which were comparable with those papers with negligible capillarity e.g. coated paper, bacterial cellulose film and weighing paper (2.2 ~ 4.1 × 10^−3^ Ω·cm).When ECA was coated on PET, it showed the highest resistivity.

**Figure 3:**
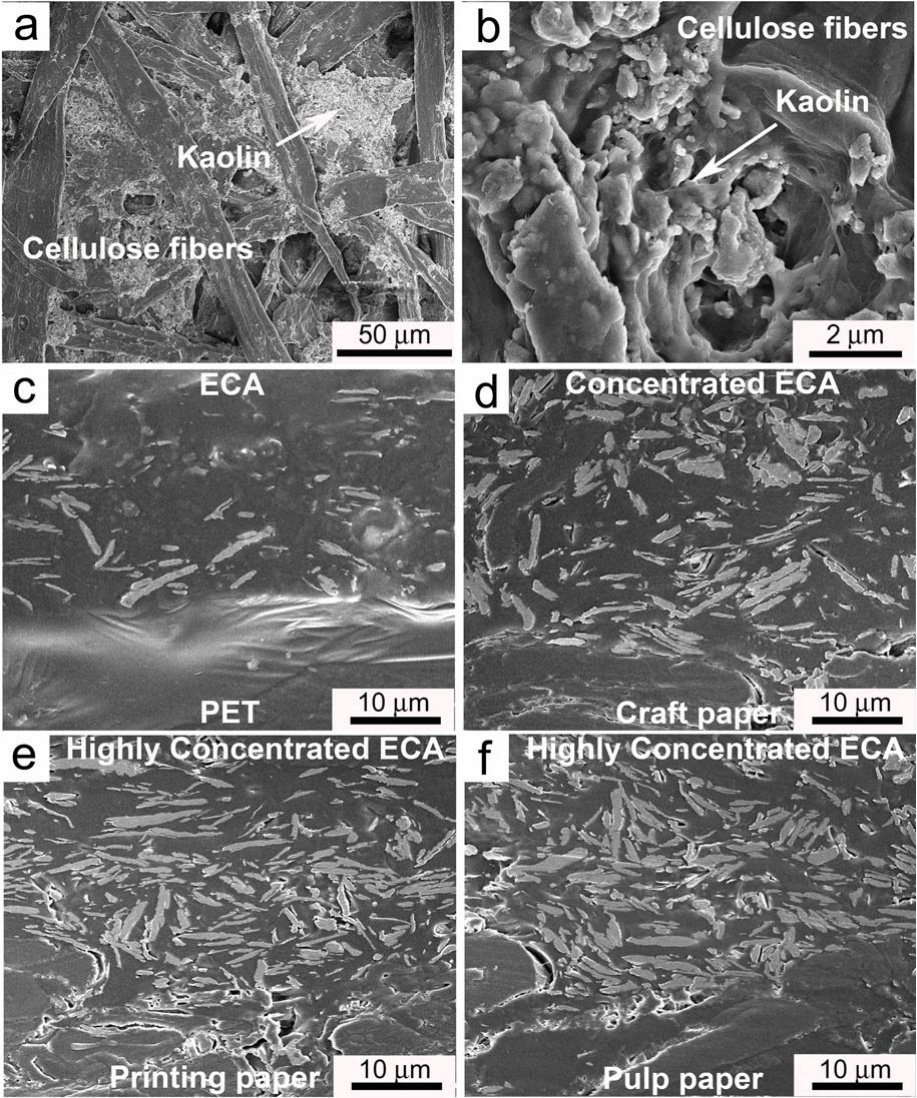
(a) (b) SEM images of the paper surface constructed by cellulose fibers and Kaolin. The Kaolin served as a sizing agent which hinders the penetration of binder. (c)~(f) Cross section at the interface between ECA (50 wt%) and substrates; From c to f, the substrates are PET, craft paper, printing paper and pulp paper; These figures clearly show that silver flakes are condensed with the decrease of sizing degree of the substrates.

It is known that the conductivity of ECAs depends not only on the filler loading but also the size, distribution, orientation and interfacial situation of the fillers^27^,^28^,^29^. A scanning electron microscopy (SEM) analysis was carried out to investigate whether the absorption process can influence the orientation of the silver micro-flake fillers. In this experiment, the SEM images of cross-section for the samples (Fig. 3 and Fig. S3) demonstrated that the silver flakes exhibit a random distribution in ECA which is printed on PET and coated paper. When the ECA was printed on papers which have medium sizing, i.e. craft paper and newspaper, the infiltration of the resin binder substantially concentrated the stacked silver flakes in ECA and then rendered a compact distribution of fillers, resulting in the enhanced conductivity. For those papers with strong capillarity such as the printing paper and pulp paper, this strong binder absorption phenomenon not only improved the contact of the fillers, which increased the probability of forming the conductive network, but also improved orientation order of the silver flakes, as shown in Fig. 3. Both of the conductivities of ECA printed on them were drastically improved.

In order to better understand the mechanism of this phenomenon, we constitute a classical dispersed phase connectivity problem and propose that the silver flakes distribute in the resin binder as a two-phase mixture. According to the standard percolation theory, we choose a well-known effective medium approximation (EMA) model for percolation theory to verify the validity of the experimental distribution obtained. Among different EMA models, the specific model we use is the modified Kirkpatrick model^30^,^31^:
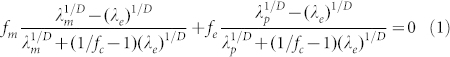
Where *λ_p_*, *λ_m_*, and *λ_e_* are the silver particle conductivity, resin binder conductivity and the overall effective conductivity, respectively. The values of *λ_p_* and *λ_m_* are estimated by common material library data. *f_c_* is the critical volume fraction of the dispersed phase, which is related to the percolation threshold of the silver particles. *D* and *f_e_* are the exponent and effective volume fraction, which are explained below. This model relates the conductivity with *f_e_* across a wide range of volume ratio. With the parameter *D*, this model is designed for systems with complex morphology, as in our case, where the silver flakes tend to orient due to the binder flows and the mixture contains non-negligible electrically resistive interfaces. *D* inherits these kinds of configurational and interfacial properties of the printed mixture. *f_c_* has been estimated from the observation of resistance transition during direct measurement of the resistance of the printed PET specimens. We use *f_e_* in equation (1) instead of *f_p_* and *f_m_*, where *f_p_* and *f_m_* are the apparent silver particle volume fraction and the apparent binder matrix volume fraction, respectively. This change takes into account of the selective capillary absorption effect of binder by paper substrates, where this absorption affects the actual volume fraction in the printed products (see Supplementary Information). We aim to quantify this effect by expecting Δ*f* = *f_e_* − *f_p_* to be realized in printed ECA products, especially observable for paper types with high capillarity. In general, a positive value of Δ*f* for a certain paper product means the corresponding amount of binder has been absorbed into the paper substrate by capillary effect. A higher Δ*f* value indicates a stronger capillary effect and vice versa.

The capillary absorption effect can be modelled as follows. Firstly, we define the absorption volume fraction *f_loss_* to be the ratio between the volume of binder absorbed inside the paper substrate and the volume of all binder in both printed ECA and paper regions, i.e. *f_loss_* = *V_loss_/V_m_*. Then we assume that, there is a characteristic balance between the capillary forces on the binder in the ECA region and the paper substrate region, and therefore maintaining a distinctive binder volume ratio between the two regions. That is, for a certain kind of ECA, *f_loss_* is a characteristic constant for each type of substrate papers. With this definition of *f_loss_*, the relationship between *f_e_* and *f_p_* can be readily derived (see Supplementary Information).

Based on the experimental data from Fig. 2a, b, and c, a theoretical best fit is carried out and we could obtain Fig. 2d. Values of *λ_m_* = 10^−10^ S cm^−1^, *λ_p_* = 1 × 10^6^ S cm^−1^, *f_c_* = 0.045 and *D* = 3.0 are used. These values are obtained from printed ECA products which has negligible capillarity (such as PET and weighing paper), i.e. *f_loss_* = 0. After the fitting procedure for the model curve parameters, the *f_loss_* for the other printed products can then be determined accordingly by a theoretical best fit of the experimental data in the established model. The subsequent *f_loss_* results are listed in Table 1. More details of the calculation procedure are discussed in electronic supplementary information.

Fig. 2d shows a fit of the experimental data with the model curve, especially in the well-percolated regime at high *f_e_* value. The high correlation of curves for different paper substrates to the model equation indicates that the proposed model can well interpret the physics inside the mixture system. The value of *D* = 3.0 deviates considerably from common value of *D* = 1.0 for spherical disperse phase, designating the particular shape effect of the silver flakes and the peculiar interfacial effects in the mixture. The modelling result suggests that the enhanced conductivity caused by the capillary binder absorption is equivalent to an increase of silver concentration in ordinary two-phase ECA mixture, and this absorption can be quantitatively determined for each paper substrate. As shown in Table 1, the stronger the capillarity of the substrate leads to higher ECA conductivity. Among all the tested substrates, the general trend of the experimental data fits the modelling equation well, and confirmed the validity of the model.

**Figure 4:**
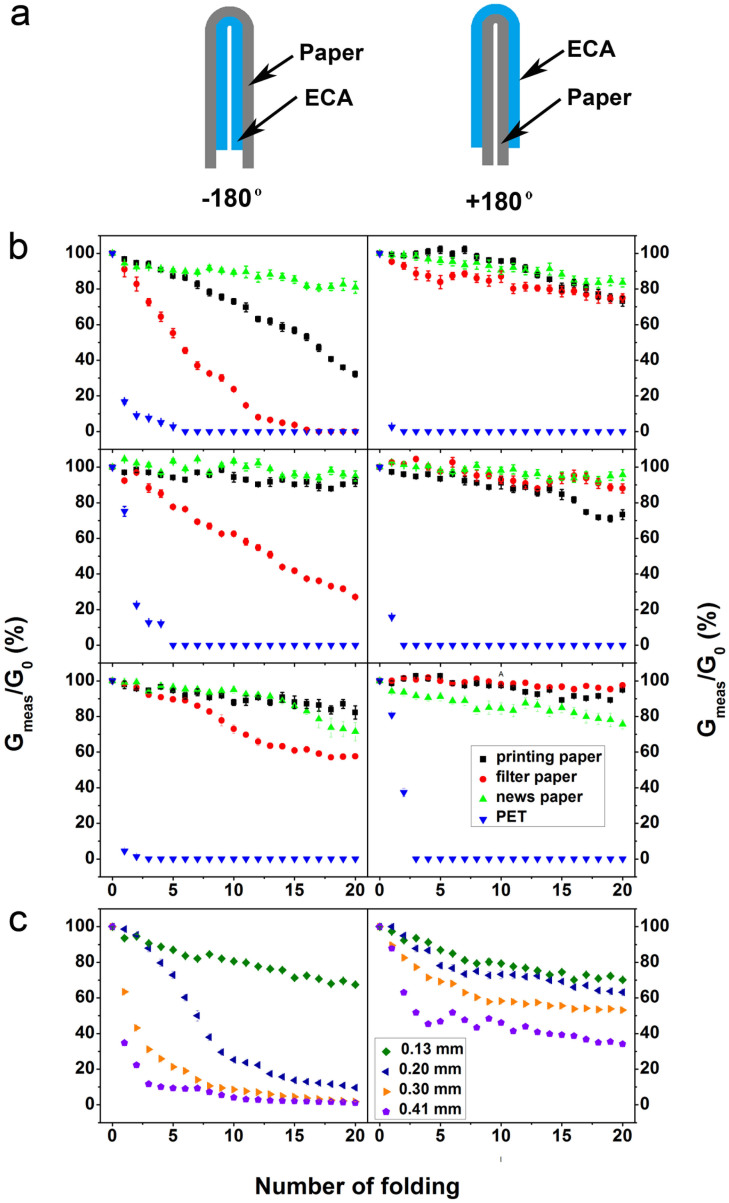
(a) A scheme of the folding test (acutely and obtusely). (b) Variation of the conductance of the ECA with different silver loading on various conductances (from top to bottom: 40, 50 and 60 wt% silver loading). (c) Variation of the conductance of the ECA on substrates with different thickness (ECA: 50 wt% silver loading).

A folding test was employed to evaluate the foldability of the samples. The test was carried out by measuring the conductivity variation after folding the papers acutely (−180°) and obtusely (+180°) for 20 cycles, respectively. As depicted in Fig. 4b, the filter paper (thickness: 150 μm) shows a faster rupture of ECA, as compared with the printing paper (thickness: 110 μm) and newspaper (thickness: 60 μm). The anti-fatigue ability of the ECA seems to depend on the thickness of the papers. A thicker paper will generate a stronger stress, and thus the ECA enduring a stronger stress shows a faster rupture. In order to describe the case systematically, we introduced pulp papers with various thicknesses as the substrate. The thickness was controlled from 0.13 to 0.41 mm by adjusting the quantity of the pulp. A thicker substrate tends to cause a serious deformation mismatch for the ECA under folding since it endures a stronger stress. Thus a thicker paper will cause a faster decrease of conductivity under folding (Fig. 4c), indicating a stronger damage of the conductive network in ECA. With the increase of the silver loading, the conductivity variation gap among the filter paper, printing paper and newspaper becomes narrower. The increased silver loading is advantageous to increase the probability of contact of the fillers so that adequate conductive pathways at the deformed area can be preserved. A considerable conductivity can be maintained after the obtusely folding test, suggesting that the property of ECA is more stable for tensile strain rather than compression.

The cross-sectional images of the folded paper with the ECA were investigated by SEM (see Fig. S5). The printed ECA on papers remained intact after being folded. Since papers are composed of cellulose fibers, the fibrous structure allows papers to be compressed in the bended zone, which effectively releases the stress; yet on those papers with a larger thickness e.g. filter paper, small cracks of the printed ECA may still form (Fig. S5a). As compared, when the ECA was coated on PET, it is difficult to maintain an acceptable conductivity, and the conductance was too low to be measured after several folding cycles, either in acute or obtuse case. This is caused by the total fracture in both acute and obtuse directions after being folded. This study shows that paper not only provides a capillary substrate to enhance the conductivity of ECA, but also gives reinforcement to resist failure, which shows a superior performance characteristic.

The reliability of paper based electronic samples under the accelerated aging condition was analyzed. The specimens were exposed in an 85°C/85% relative humidity (RH) environment and the resistances were recorded (see Fig. S6). Experimental results suggest that the resistivity of ECA maintained stable after 800 hours. And then an increase of around 20% was observed. The main conductive property of the ECA was preserved after the reliability test. After this harsh reliability test, the paper based thermoplastic ECA can be regarded sufficiently reliable for many electronic applications. Additionally, the mechanical interconnecting property towards the electronic components was investigated by both the lap shear test and the tape test (Fig. S7). The ECA-paper interface is even mechanically stronger than the fracture strength of paper substrate itself. Results showed that the current ECA formulation is good enough for the paper based electronic applications. A detailed comparison of the current work and the recently reported results e.g. metalized paper conductor are included in the electronically supplementary information (Table S2).

**Figure 5:**
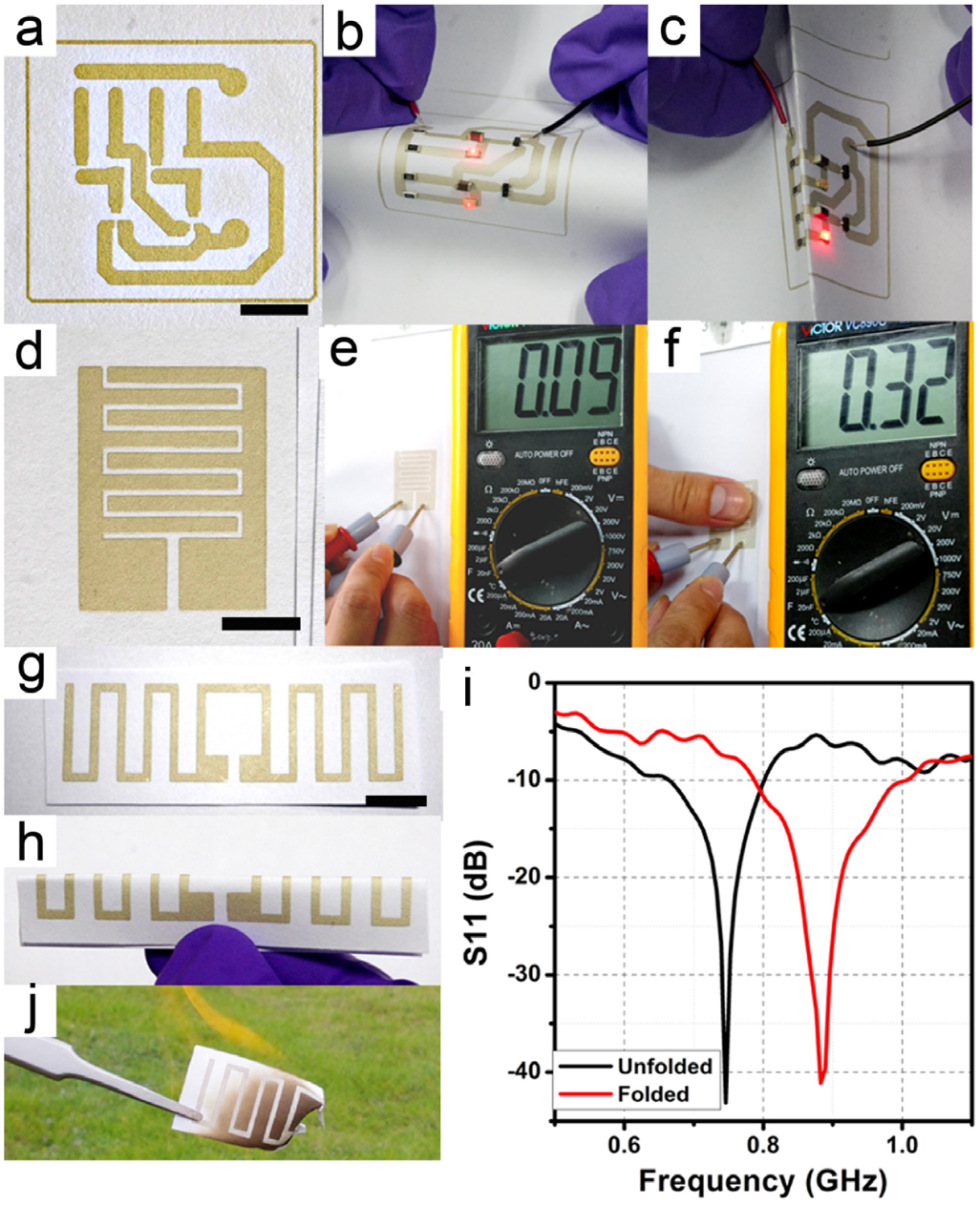
(a) A prototype of the paper based PCB for preparing an LED array winking circuit. (b) Working circuit with the placement of the necessary electronic components on the PCB. (c) Working circuit is being folded. (d) A prototype of paper based capacitor. (e) The capacitance of this capacitor without finger-touch. (f) The capacitance of this capacitor under finger-touch. (g) The prototype of paper based antenna. (h) The antenna in folding circumstance. (i) Operating frequency of the antenna measured in unfolded and folded circumstance. (j) Optical photograph of a piece of burning printed devices. The scale bars in the figures are 1 cm.

In order to demonstrate the practical application of the current technique, we carried out a series of experiments to evaluate the effectiveness of the prototypes. Three prototypes included a light emitting diode (LED) winking device, a capacitive touch pad, and an ultra-high frequency-radio frequency identification (UHF-RFID) antenna. All of them were prepared by screen printing ECAs (50 wt% of silver) on paper. As shown in Fig. 5a~c, the LED winking device is fabricated by mounting the electronic components on a paper based substrate. This kind of device is able to provide the function of lighting instructions in public signage, toys or advertisements. The switching of the LED is determined by adjusting the base voltage of the transistor which is controlled by charging a capacitor. As can be seen in Video S1 and S2; charging and discharging two capacitors resulted in the winking of two LEDs alternatively when the circuit was connected to a DC source (3 V). The device worked well even when it underwent bending or folding circumstance.

Fig. 5d~f illustrates the function of the paper based capacitive touch pad which showed a capacitance of 0.09 nF without finger-touch. Since human body has some body capacitance (~100 nF), when a finger touches this capacitive touch pad, the recorded capacitance increases. This increment is detectable and potentially available to make a green and disposable button for paper based touch panels^6^. We printed the same layout on various substrates, and the optical images were presented (see Fig. S8). The printed capacitors on paper exhibited a capacitance around 0.1 nF and they were enhanced to 0.3 nF by a finger-touch. As compared, the printed capacitive touch pad on PET only showed 0.05 nF and 0.18 nF by finger-touch. Since cellulose possesses higher dielectric constant than PET, the separated conductive plates by cellulose mainly contributes to the higher capacitance of the paper based devices^32^. Moreover, the silver flakes are more densely packed on paper rather than on PET, thus charge can be accumulated on the conductive plates. This may also contribute to the high capacitance. The result indicates a better performance of printed devices on papers rather than the control sample on PET.

In addition, we also evaluated the effectiveness of the printed ECA on paper as foldable RF-devices, in order to demonstrate the potential application on portable wireless communications or label identifications. As shown in Fig. 5g~i, the operating frequency of the parameter of the printed antenna sample centred around 741 MHz in the unfolded status. This operating frequency of the antenna shifts to 883 MHz when it was folded along the middle line of it, which shows a distinctive response when folded. The return loss of S11 is below −40 dB in both cases, indicating a well-match of the antenna to the cable. This signal response of the antennas suggests an excellent performance of the printed RF-device on paper for practical applications. Besides, the antennas were also printed on various substrates and the optical images are shown in Fig. S9. The operating frequency of antennas varies from 694 to 762 MHz. This variation may be related to the different dielectric constants and thicknesses of the substrates. The antenna printed on PET exhibit a return loss of −22 dB, which was relatively weaker among the specimens. Although the response of antenna is controlled by a few electromagnetic factors of the whole system, the relatively large resistance can be regarded as an adverse effect to the real part of impendence, which may render impedance-mismatch, and then results in a weakened response of the antenna to the signal. When these paper based RFID antennas are disposed and become e-waste, paper-based ones can be easily degraded by such as a simple burning process and the silver remnant is able to be recycled, as shown in Fig. 4j^33^. Based on the above experiments, it appears that the paper based electrical devices are superior to their counterpart based on PET.

## Concluding remarks and outlook

In summary, we have successfully demonstrated that by taking the advantage of the capillary characteristic, ordinary paper substrates can excellently match with the thermoplastic ECA to achieve superior electrical conductivity, mechanical strength and reliability as compared with the non-porous plastic substrates. By taking into account a series of characteristics of a few kinds of papers, we selected sizing degree as the main factor for controlling the enhancement of electrical conductivity of the printed ECAs. Based on a modified Kirkpatrick model that we established, the resin binder taken up by both the paper substrates and the ECA composite shows a characteristic volumetric ratio, which follows the capillary balance between the two regions. Based on a curve fitting procedure for the conductivity curves, we quantified the binder absorption for each paper substrate under the same theoretical effective medium model. Moreover, due to the reinforced interface, the ECA tracks printed on the papers can be folded repeatedly with negligible mechanical and electrical loss. We also demonstrated some basic applications of the new materials in circuit boards, capacitive touch pads and antennas for future green electronics.

Nevertheless at this stage, a few technical issues, e.g. fire-retardancy and a smoother surface, stable insulation and dimensional reliability of the paper substrate need to be addressed before their practical applications in industry;since paper substrates are much more economical and are more readily renewable materials when compared to common circuit board substrate materials, we believe that the current method may soon find broad applications in consumer electronics which welcome low cost, eco-friendliness, recyclability, and flexibility. We look forward to the accomplishment of the related techniques and broader application of this new technology.

## Methods

### Preparation of materials

The paper substrates include commercial printing paper (Fuji Xerox), filter paper (Chunghwa Double Rings), pulp paper (Bleached eucalyptus pulp, provided by Guangdong Ding-Feng Pulp-Making Co., a holding company by Chung Hwa Pulp Co.), newspaper, the recycled paper from the paper pulps and the coated paper. The pulp and recycled pulp paper were obtained by filtering the raw wood pulp through a plain cotton cloth followed by drying and pressing processes. The thickness of most paper is about 100 micron. Table 2 shows the basic physical properties of some ordinary papers. PET films (Dupont-Tijin Melinex EL) were used as the control substrates. The sizing degree is the ability to reduce the absorptivity of water or other fluids of the paper, which is described as the time the liquid is totally absorbed by the paper; and this characteristic is related to a variety of the properties of the paper, including the property of the cellulose fiber, pore size, surface roughness, and the content of other additives etc. The sizing degrees of the filter paper, pulp paper and recycled paper were tailored by coating polyvinylalcohol (PVA) and bacterial cellulose fibers, and were carefully measured by the absorption time of a liquid in a closet with stable temperature and humidity. The ECA sample was composed of silver flakes (Changcheng Bank Note SF01A) and a thermoplastic resin binder (pBMA-co-MMA, DSM B-725). Cyclohexanone (Sinopharm Chemical Regent Co., Ltd) was used as the solvent. The loading of ECA is adjusted by controlling the ratio of silver flake to binder. The fillers, binder and a certain portion of solvent were mixed in a planetary rotary mixer (Hasai Co., Shenzhen) with 1,500 rpm for 5 minutes to form a homogeneous paste. Then the ECA paste was screen-printed onto the paper substrates before being dried at 80°C for 15 minutes in a memmert oven.

### Characterization

The morphologies of the surface of various papers and the cross-section of ECA on papers were investigated using a field emission scanning electron microscope (FE-SEM) (Hitach S-4800). Prior to the measurement, the cross sections of the specimen were embedded in phenolic molding compound and microtomed by glass knife. The sizing degree of papers was determined by measuring the time that a droplet (~0.02 g) of ECA's binder (the ratio of pBMA-co-MMA to cyclohexanone was 1:2 by weight) was totally absorbed by a paper. The bulk resistance of the film was measured by the four-point probe method with a multimeter (SANWA CD800a, Japan); the thickness of the ECA on paper was regarded as the same as the one on PET which was measured as the distance between the top of ECA and the surface of PET with a micrometer (Shenliang Precision Measuring Co. Ltd., China). Five values were recorded and averaged to obtain the thickness value. Then the volume resistivity can be observed as: *ρ* = *R* × *w* × *t/L*, where *ρ*, *R*, *w*, *t* and *L* represent the resistivity, bulk resistance, width, thickness and the length of the printed conductive track on papers, which was determined by the sample geometry on a non-absorptive PET. The reliability test of ECA on papers was carried out by aging the specimen into a chamber (ESPEC SETH-Z-042L). The condition in the chamber was maintained at 85°C/85% relative humanity for 1,000 hours. The capacitance of the printed capacitive touch pad was measured by a multimeter (Victor VC890C+), and the return loss of the printed antenna was detected by a network analyzer (HP 8753D).The lap shear strength test was carried out on a MTS Universal Testing Machine (SUNS-6104). Prior to the test, the ECA samples were filled in the gap between two substrates, and the dimension was carefully controlled to be 10 × 10 × 0.06 mm^3^. After that, the specimen was tailored in 110 mm length and 10 mm width (as shown in Fig. S5a). Then two substrates were stretched in the opposite direction with the speed of 2 mm/min until the specimen broke. The tape test was carried out by pasting and peeling a tape (3 M) on the paper based ECA.

## Author Contributions

H.W. and C.Y. wrote the manuscript, performed the experiments. S.C. finished the simulations. C.Y. conceived the concept and revised the manuscript. W.L., Z.L., J.L., X.C., F.K. and C.W. reviewed the manuscript.

## Supplementary Material

Supplementary Information Electronic supplementary information

Supplementary Information ESI video#1

Supplementary Information ESI video#2

## Figures and Tables

**Table 1:** *f_loss_* ofvarious paper substrates estimated from the model established using non-capillary substrate. A higher *f_loss_* value for a substrate indicates higher capillary absorption ability. The conductivity of ECA with 50 wt% of silver filler is listed to show the benefits from binder absorption quantitatively

Papers	Sizing degree (s)	*f_loss_*	*λ_e_*(×10^3^ S·cm^−1^)
Pulp paper	~3	0.70	16.1
Filter paper	~3	0.67	14.3
Recycled pulp paper	~9	0.63	7.7
Printing paper	~16	0.62	10.0
Newspaper	~25	0.49	3.4
Craft paper	~35	0.51	6.7
Coated paper	~2380	0.20	0.5
Weighing paper	~3120	0.10	0.2
PET	---	0.00	0.2

**Table 2:** Comparison of the physical properties of some papers

	Thickness (micron)	Cellulose content (wt%)	Tensile strength (MPa)	Young's modulus (MPa)	Roughness (micron)	Price (US$/ton)Sources
Coated paper	~400	~70	2.16	19.82	~0.3	~1100
Printing paper	~110	~76	2.92	10.01	~1.7	~1200
Filter paper	~150	~89	0.77	3.72	~5.3	>3000
News paper	~60	~80	2.93	3.78	~2.4	~340
Weighing paper	~30	~92	3.73	2.56	~0.9	>3000
Craft paper	~370	~83	2.43	14.97	~2.7	~1400
Tissue paper	~40	~91	0.26	0.12	~8.3	~1800
Pulp paper	-	~94	0.33	33.20	~4.8	-
