# Supplementary material for: Towards Practical Application of Paper based Printed Circuits: Capillarity Effectively Enhances Conductivity of the Thermoplastic Electrically Conductive Adhesives
Source: Sci Rep. 2014 Sep 3;4:6275. doi: 10.1038/srep06275 (PMC4152754; doi:10.1038/srep06275)
Supplement: Supplementary Information — Electronic supplementary information [file srep06275-s1.doc]

**Electronic Supplementary Information**

Towards Practical Application of Paper based Printed Circuits: Capillarity Effectively Enhances the Conductivity of Thermoplastic Electrically Conductive Adhesives

Haoyi Wu ^1^, Sum Wai Chiang ^1^, Wei Lin,^2†^ Cheng Yang*^1^, Zhuo Li ^2^, Jingping Liu ^1^, Xiaoya Cui ^1^, Feiyu Kang^1^, Ching Ping Wong^2,3^

^1^Division of Energy and Environments, Graduate School at Shenzhen, Tsinghua University, Xili University Town, Shenzhen City, China.

Fax: +86-755-2603-6417; Tel: +86-755-2603-6132; E-mail: yang.cheng@sz.tsinghua.edu.cn

^2^School of Materials Science and Engineering, Georgia Institute of Technology, 771 Ferst Drive, Atlanta, GA, 30332, USA

^3^Department of Electronic Engineering, Chinese University of Hong Kong, Shatin, NT, Hong Kong SAR, China

† Current address: IBM Albany Nanotechnology, 257 Fuller Road, Albany, NY, 12203

**Contents**

[Resin binder characteristics 3](#__RefHeading___Toc385615515)

[Printing resolution 3](#__RefHeading___Toc385615516)

[Thermogravimetric analysis of papers 3](#__RefHeading___Toc385615517)

[SEM analysis of the ECA samples on various substrates 5](#__RefHeading___Toc385615518)

[Modelling method 7](#__RefHeading___Toc385615519)

[Photography of the folded samples 11](#__RefHeading___Toc385615521)

[Reliability test 13](#__RefHeading___Toc385615522)

[Lap shear test and tape test 14](#__RefHeading___Toc385615523)

[Demonstration of printed capacitors and antennas 15](#__RefHeading___Toc385615524)

**Resin binder characteristics**

The poly(BMA-co-MMA) copolymer resin for the binder of ECA is soluble in many solvents. In addition, the glass transition temperature and the molecular weight of the copolymer is 63^o^C and 55,000 respectively, which demonstrate the softness and availability for screen printing.

**Printing resolution**

In order to evaluate the feasibility of printed electronic on paper, the ECA is screen printed onto a common printing paper (Fuji Xerox) as shown in Figure S1. The resolution of ECA printed on paper strongly depends on the surface roughness of paper and the printing method. Using screen printing, the finest lines of printed conductive track on the printing paper was able to reach 0.2 mm, and the resistance of the line was maintained steady. We believe that a smoother surface can further improve the resolution of the fine structure. The limitation of further improvement may be related to the selection of printing method.


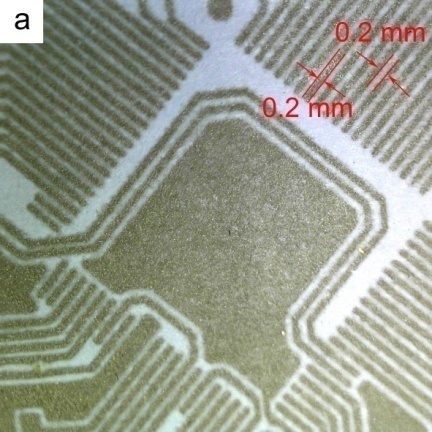

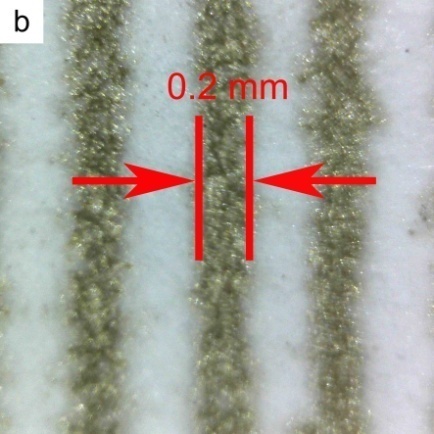


Figure S1 Optical image of the ECA screen printed on printing paper. The width of the printed conductive line can be minimized to 0.2 mm, which is promising for many electronic applications.

**Thermogravimetric analysis (TGA) of papers**

In order to estimate the cellulose content of various kinds of paper, the thermogravimetric analysis was carried out. As exhibited in Figure S2, the pulp paper which is regarded as composed of 100% cellulose, lost almost 100% weight at around 500^o^C, indicating the completed decomposition of cellulose fibers. As can be seen, the tissue paper, weighing paper and filter paper contain more than 95 wt% cellulose. The newspaper and craft paper contain around 90 wt% of cellulose, and the printing paper and coated paper contain 78 and 75 wt% cellulose respectively. The mass loss of paper higher than 500 ^o^C is attributed to the bulking agent and sizing agent *etc*. In fact, the sizing degree of weighing paper is strong (regarded as nearly no capillary force) due to the surface sizing agent --- paraffin wax, which may simultaneously loss the weight during the heating process. The sizing degree of printing paper is strong to water yet it is weak to the pBMA/MMA-cyclohexanone binder since it only takes around 16 seconds to penetrate a droplet of the latter. It is to be noted that the weight loss below 100 ^o^C is primarily due to the elimination of the absorbed water molecules.


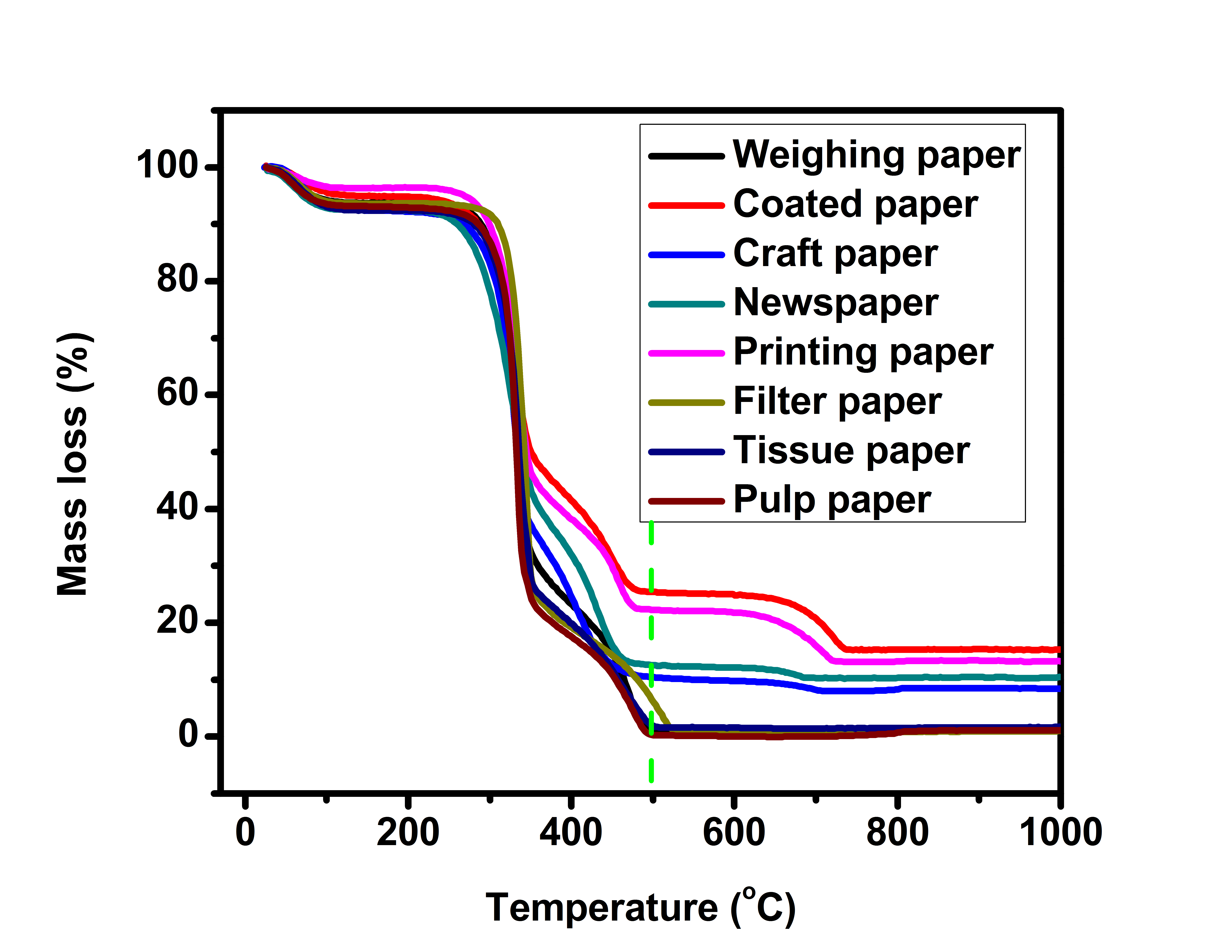


Figure S2 A TGA spectrum of various kinds of paper; the pulp paper lost almost 100% weight at 500 ^o^C, indicating that cellulose fiber completely decomposed. The cellulose content can be roughly estimated by monitoring the lost weight of paper between this temperature and 100 ^o^C.

**SEM analysis of the ECA samples on various substrates**

Figure S3 shows the SEM images of the ECA samples. As can be seen, for the case of PET substrates, the silver flakes in ECA (50 wt% of silver) distribute randomly. For the paper substrates with a strong sizing degree, e.g. coated paper, the capillarity is greatly suppressed. Hence the infiltration ability of the resin binder is strongly reduced. Only a small portion of the resin binder is absorbed by paper, and thus the flakes are sparsely dispersed in the resin matrix as shown in Figure S3 c. When ECA is coated on the craft paper and newspaper with medium sizing degrees, the infiltration of the resin binder substantially concentrates the stacked silver flakes in ECA on top of the paper. Then the shortened distance between silver flakes (Figure S3 d and e) leads to a substantial decline of electrical resistivity. For papers with a weak sizing effect, i.e. printing paper and filter paper, this situation is more significant. As presented in Figure S3 f and i, this absorption not only provides an intimate contact of fillers, but also induces some orientation of the silver flakes *via* compressing the space. The alignment of silver flakes further reduces the resistivity of the ECA. With the increase of silver loading, the intimate distribution accompanied by the alignment of silver flakes reduces the resistivity of ECA accordingly. Figure S3 g~k show the interface between filter paper and the ECA with various silver loading. The increased loading causes a high volume fraction of silver flakes and also reduces the resistivity, which suggests the enhanced conductivity by capillarity equivalent to that by increasing filler loading.


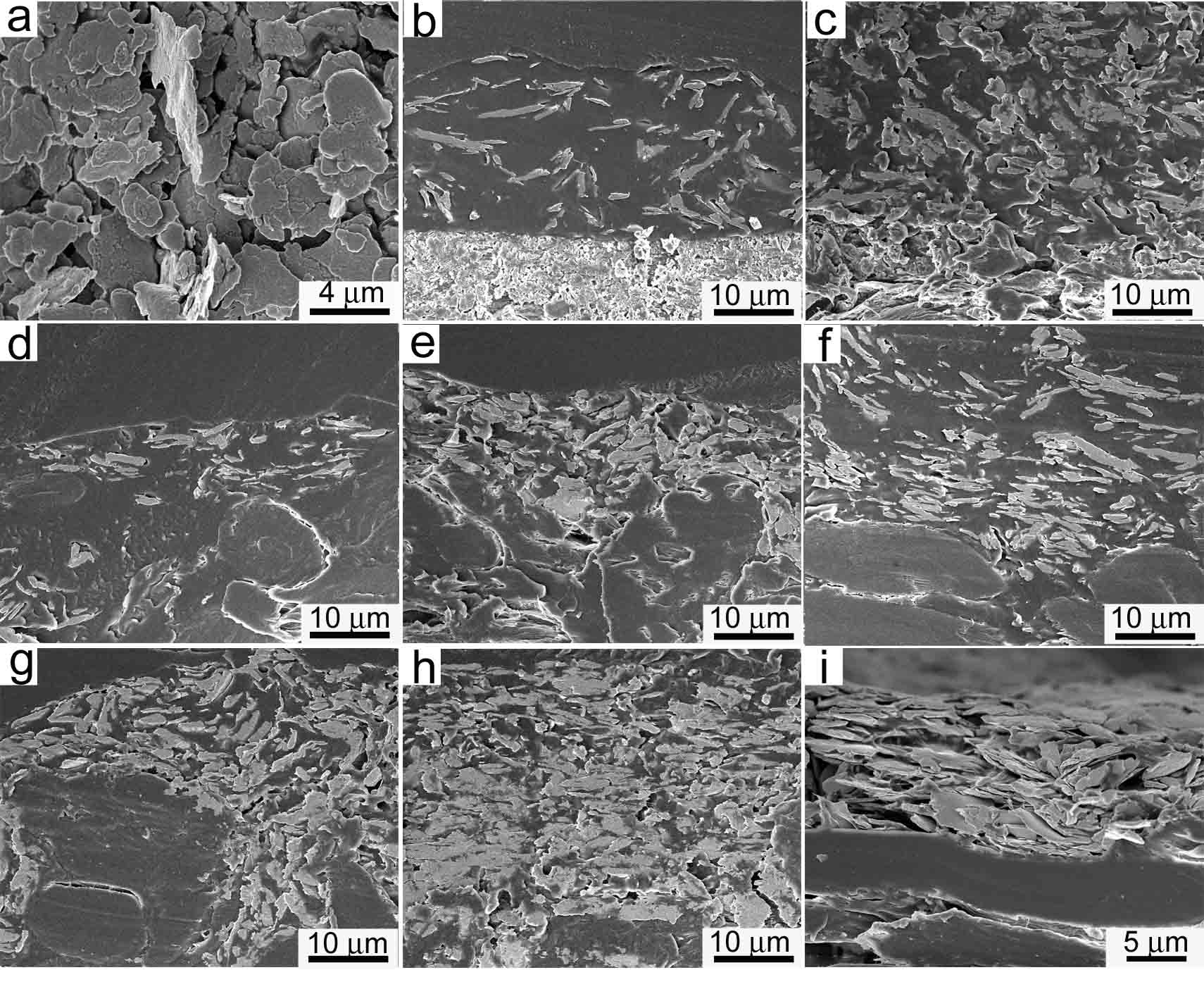


Figure S3 (a) SEM images of the silver microflakes employed as fillers for ECA; b~i: SEM images of the cross section at the interface between ECA and various substrates, b: coated paper and ECA with 50 wt% fillers, c: newspaper and ECA with 50 wt% fillers and d~h: filter paper and ECA with 30, 40, 50, 60 and 70 wt% fillers; i: Cross-section of ECA (50 wt%) screen printed on a printing paper. The images clearly show the silver flakes are condensed with the decrease of sizing degree of the substrates, as well as the increase of the silver loading. It agrees well with the demonstration about Figure 3. Besides, an obvious face-to-face alignment of silver flakes can be observed by screen printing.

**Modelling method**

Table S1: Nomenclature

| *f_p_, f_m_*  *f_c_*  *f_e_*  *λ_p_, λ_m_*  *λ_e_*  *V, V_p_,V_m_,V_loss_*  *D*  Δ*f*  *f_loss_* | Volume fractions of silver particles and binder matrix.  Critical volume fraction, correspond to the percolation threshold.  Effective volume fraction of silver particles, considering the absorption effect of paper substrate.  Conductivities of silver particles and binder matrix (S·cm^-1^).  Effective conductivity of the overall composite on the paper substrate (S·cm^-1^).  The volumes of the whole ECA, all silver particles, all binder, and binder being absorbed by paper substrate after application on paper (cm^3^).  Inverse of exponent, representing the bulk effects from interfaces and contacts.  The difference *f_e_*– *f_p_ .*  A volume fraction defined as *V_loss_* / *V_m_ .* |
| --- | --- |

In order to compute the theoretical percolation trends of the ECA, we employed the modified Kirkpatrick model (equation 1) as our basis for comparison using the nomenclature as shown in Table S1. The model is based on network connection theory and is built with the considerations of microstructure percolation and interfacial properties of the particles. This model is suitable for systems with complex morphology as in our case, where the silver flakes tend to be oriented strongly due to the binder flows and the mixture contains non-negligible electrically resistive interfaces.

The percolation of particles is controlled by the critical volume fraction *f_c_* in the equation, and the interfacial and orientation of the particles are controlled by the exponent inverse *D*. The *D* inherits this kind of configurational and interfacial properties of the printed mixture. The *f_c_* has been estimated from the observation of resistance transition during direct measurement of the resistance of the printed specimens. We use the *f_e_* in equation (1) instead of *f_p_* and *f_m_*. This change put into consideration the selective capillary absorption effect of binder by paper substrates, affecting the actual volume fraction in the printed products. We aim to quantify this effect by expecting a Δ*f* = *f_e_*– *f_p_* to be realized in printed ECA products, especially for paper types with high capillarity. In general, a positive value of Δ*f* for a certain paper product means the corresponding amount of binder has been absorbed into the paper substrate by capillary effect. A higher Δ*f* value indicates a stronger capillary effects and vice versa.

The definition *f_loss_*= *V_loss_/V_m_* is a characteristic ratio meant to be constant for a certain pair of paper versus ECA. After some straight forward algebra, it is easy to obtain the following relation from the definitions above. Herein,

$f_{p}=\frac{V_{p}}{V}$ (S1)

$f_{m}=\frac{V_{m}}{V}$ (S2)

and $f_{e}=\frac{V_{p}}{V-\Delta V}$ (S3)

then , we have $Vf_{e}-V_{p}=\Delta Vf_{e}$ (S4)

We define, $f_{loss}=\frac{\Delta V}{V_{m}}=\frac{Vf_{e}-V_{p}}{f_{e}f_{m}V}=\frac{f_{e}-V_{p}/V}{f_{e}f_{m}}=\frac{f_{e}-f_{p}}{f_{e}f_{m}}$ (S5)

then $f_{loss}=\frac{\Delta f}{f_{e}f_{m}}$ (S6)

Given equation (S1), the value of *f_e_* required in equation (1) (in the main text) can be easily obtained from the *f_p_* value as follow

$f_{e}=\frac{f_{p}}{1-f_{loss}f_{m}}$ (S7)

Given *f_c_*, *D*, *λ_m_* and *λ_p_* as parameters, the relationship between *f_e_* and *λ_e_* can then be plotted. The parameters *f_c_* and *D* are unknown in advance since they depend strongly on the silver particles' and paper substrates' properties. In this work, we find their values by least-square fitting the two parameters to a subset of experimental data, as can be elucidated below. Another choice we have made is to use *f_e_* instead of *f_p_* , and the reason is the existence of selective absorption of the binder by the paper substrate, resulting in an increase of volume fraction of silver on the mixture formed on substrate surface, and this value is described as *f_e_*.

The experiments on the ECA are conducted on PET surface first, which is free from binder absorption. In this case, we know *f_e_* = *f_p_*, and the resulting measured conductivities versus *f_e_* are fitted to the theoretical curve by least-square determination of the parameters *D* and *f_c_*. Then we assume the change of these two parameters should be negligible as long as the same silver and binder materials are applied. We assume the use of different paper substrates only affect the value of *f_loss_*. Using the established curve, we estimate the value of *f_loss_* on each of the paper substrates by least-square fitting the experimental data to the curve. The *f_loss_* determines the Δ*f* according to equation (2). Therefore, from this procedure, we can determine quantitatively the value of *f_e_*, once given a *f_p_* value.

Figure S4 shows the fitting process graphically. As shown in Figure S3 (a), the experimental data obtained on PET are used to establish the model curve first. Then, other data points are least-square fitted onto the established curve. We notice that all the data points are on the left side of the theoretical curve. This is in accordance to expectation, since paper substrates absorb binder, which suggests *f_loss_* is always positive and hence a positive Δ*f* according to equation (2). By theoretically fitting the result points of each paper substrate to the calibrated curve, the process can give the *f_loss_* values which we are interested in.


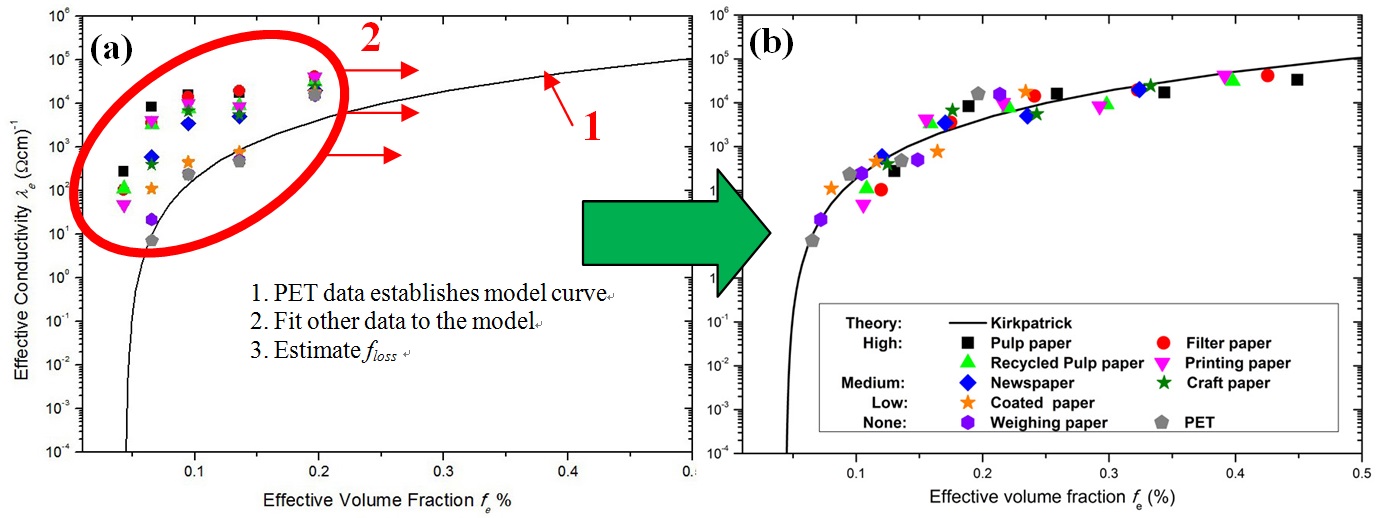


Figure S4: The data fitting process, where (a) is plotted if all *f_loss_* = 0, and (b) is plotted with least square fitted *f_loss_* values on each type of paper substrates. First, the PET data is used to establish the model curve, then other data points are least-square fitted onto the curve. This process suggests the *f_loss_* value which we are interested in.

**Photographic images of the folded samples**

The optical photography of the ECA after being folded for 20 cycles is shown in Figure S5 a. An obvious crack is observed of ECA on PET, indicating the fracture of the conductive network. However, when the ECA on a paper is being folded, the porous structure of the paper is advantageous to release the stress so as to prevent the conductive network from damaging in ECA. This is confirmed by the cross-section SEM image of the ECA under folding. Therefore, the paper based ECA presents flexible and foldable features.


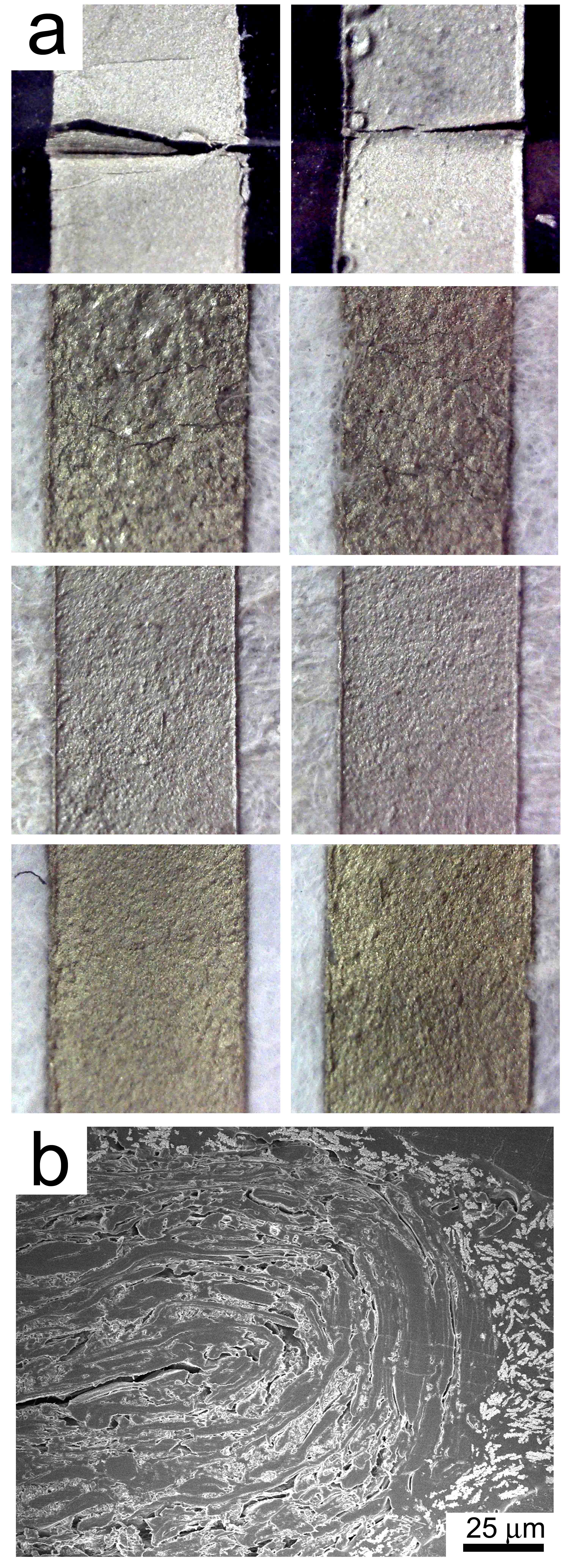


Figure S5 (a) Optical photography of the deformation of ECA on various substrates after folding 20 cycles (from top to bottom: PET, filter paper, news paper and printing paper, left and right row correspond to -180^o^ and +180^o^ folding case, respectively). The ECA on PET fractures after folding several cycles, whereas it maintains conductive on paper substrates. (b) Cross-section of the ECA (50 wt%) on printing paper under folding circumstance. The conductive network can be well preserved in ECA.

**Reliability test**

The reliability of the ECA was estimated by an 85^o^C/85%RH (relative humidity) aging test. The conductive track samples printed on a printing paper were exposed to such a circumstance for 1,000 hours. Prior to this test, the solvent in ECA was totally evaporated and the resistance of the specimen maintained constant. The resistivity variation of the ECA is shown in Figure S6. During the first 800 hours, the resistivity remained constant and then there was a slight increase (20% increment). Although the resin binder of ECA is a thermoplastic polymer with a glass-transition temperature of 63^o^C, above which the mobility of the molecular chain of the copolymer is enabled, the exposure to 85^o^C still prevent the resistivity of ECA from changing significantly, verifying excellent reliability. When the aging time is more than 800 hours, a slight enhancement of the resistance was observed, indicating the increase of instability which somehow enhances the contact resistance among the silver flakes. This experiment suggests that the reliability of the ECA samples printed on paper is well acceptable.


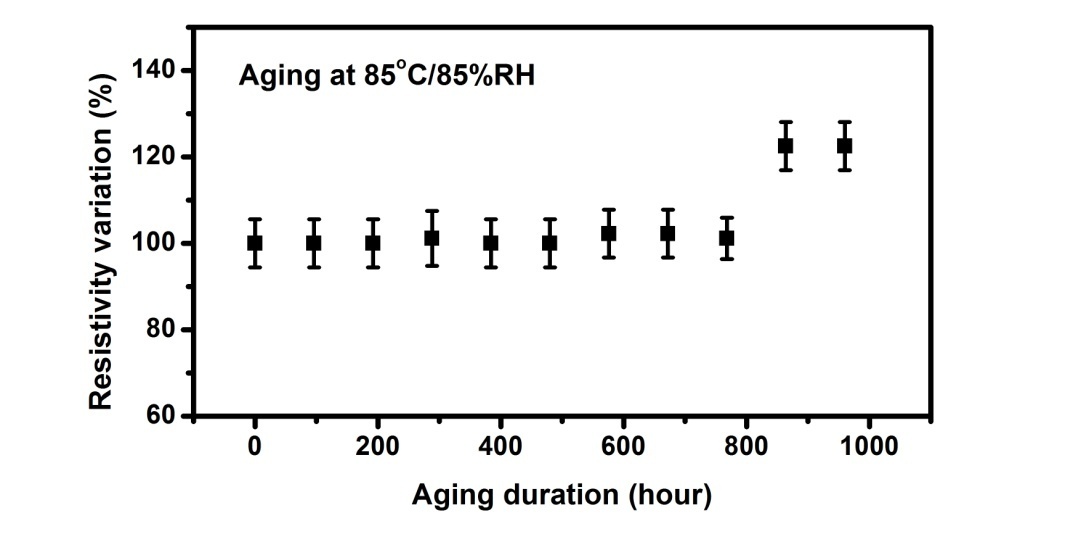


Figure S6 Reliability test of the ECA printed on a printing paper; the resistivity keeps steady in the first 800 hours and rises slightly.

**Lap shear test and tape test**

Besides electrical conductivity, mechanical property is equally important for the ECAs. In order to evaluate the adhesion of ECA on various substrates, the lap shear test and tape test were carried out. The corresponding force - displacement curves were presented in Figure S7. In the case of PET substrate, the ECA connection fractured when the specimen underwent a tensile strain at 4.2 N. The shear strength at break for the ECA was around 6.9 MPa. Instead of the disconnection at the ECA site, the fracture took place at paper substrates when paper based specimens experiencing the tensile strain. This implies that the shear strength of ECA on paper is even stronger than the fracture strength of paper. This is confirmed by the tensile strength test of paper listed in Table 1. All of them present a value smaller than 6.9 MPa. The strong adhesion between ECA and paper is further confirmed by the tape test. Even though a 3M tape is pressed on the ECA, the integrity of the samples was preserved after peeling off the tape. Therefore, unless stronger paper is designed for this special use, the adhesion strength for the current ECA seems to be adequate for the paper based electronic applications.


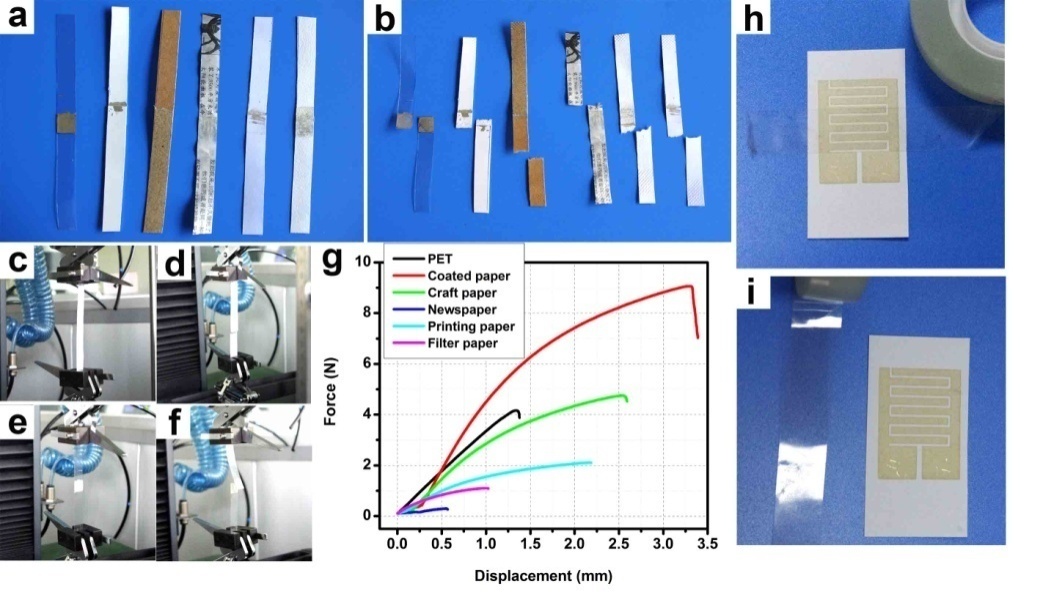


Figure S7 (a) Optical photography of various kinds of paper prior to lap shear strength test; (b) Optical photography of various kinds of paper after lap shear strength test; (c) and (d) Optical photography of two adhered printing papers before and after the test; (e) and (f) Optical photography of two adhered PETs before and after the test; (g) Force - displacement curves of the samples. The fracture of papers instead of ECA indicated that we should focus on the mechanical property of paper itself rather than ECA when we consider the mechanical properties of paper based ECA. (h) A photography which shows a 3M scotch tape attached onto the sample; (i) A photography which shows the sample after peeling off the tape. The tape test confirms the strong adhesion of the ECA on paper

**Demonstration of printed capacitors and antennas**

In order to investigate the extensiveness of printed electronics, the capacitor and antenna prototypes were printed on various substrates, and the photographic images and performances are depicted in Figure S8 and S9. As can be seen, the devices can be screen printed on the substrates very well, implying that the printed ECA is not selective to substrates, and it is available to many papers. The paper based printed capacitive touch pad is composed of two conductive plates printed on cellulose. The conductive plates serve as both electrodes and conducting traces which are connected to external electronics. The high capacitance of the paper based prototype benefits from the high dielectric constant of the cellulose, rather than that of PET. When a finger touches the gap between two electrodes, it bridges two electrodes and makes a coupling between the intrinsic capacitance from printed device and the body capacitance from human. This is reflected by the enhanced capacitance under a finger-touch. In addition, the paper based printed antennas are composed by a conductive line and a paper substrate. The shape of the conductive line and the dielectric constant of the substrate can strongly influence on the performance of the antenna. The return loss of the antenna reflects how well the device is matched. Usually, a high return loss is desirable for well matching. Our printed antenna is designed to match a 50 Ω impedance, and all of them are acceptable since they show promising values of return loss. The printed antenna on paper shows a better impedance-match than the one on PET. This may attribute to the higher conductivity of ECA which reduces the real part of the real part of impendence. In summary, it appears that the printed devices show better respond on papers rather than PET. This better confirms the advantages of printed ECA for paper based electronics.


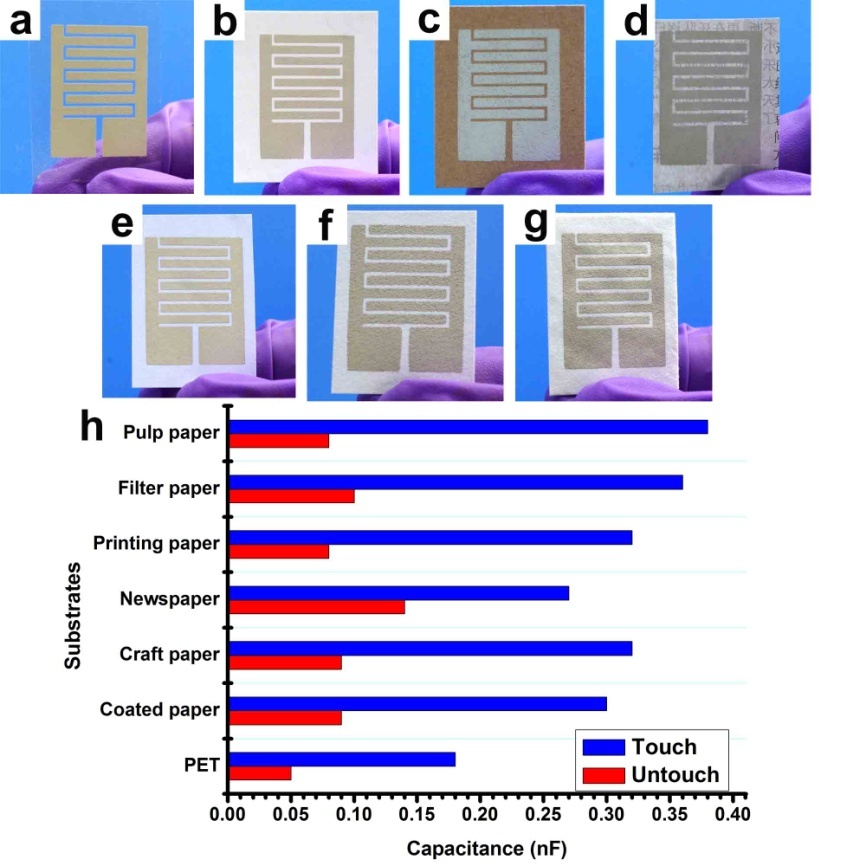


Figure S8 (a) ~ (g) Optical photography of printed capacitor on various substrates (from a to g, PET, coated paper, craft paper, news paper, printing paper, filter paper and pulp paper); (h) the capacitance at free space and under pressing. The capacitor shows a smaller respond on PET rather than papers. This may attribute to the small conductivity of ECA printed on PET.


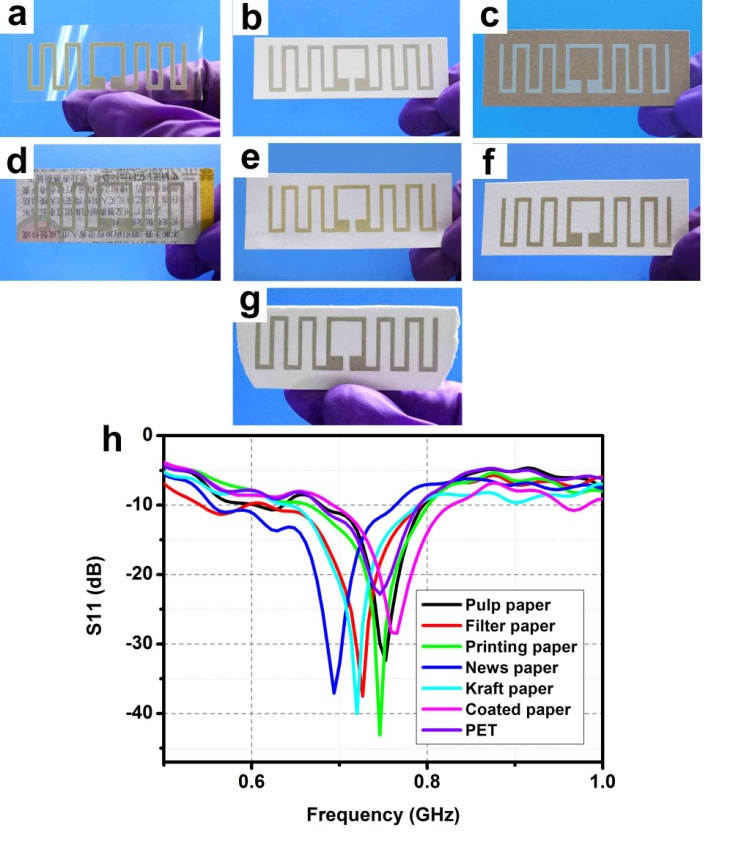


Figure S9 (a) ~ (g) Optical photography of printed RFID antenna on various substrates (from a to g, PET, coated paper, craft paper, news paper, printing paper, filter paper and pulp paper); (h) The corresponding return loss of S11 of the printed antennas. The antenna printed on PET shows the smallest return loss. This is ascribed to the impendence mismatch caused by the smaller conductivity of ECA printed on PET.

Table S2 lists the recent reports on paper based electronics, including direct-written conductive inks, sputtered or evaporated metals, desktop-printed liquid metal and ink-jet printed conductive inks. All these methods have successfully attained conductive networks on paper. Compared to pure metal and the sintered nanoparticles, ECA does not show a superior conductivity because of the contact resistance in the composite. However, the binder absorption of paper renders it an enhanced conductivity (in the range of 10^-5^ Ω﹒cm) which is feasible in many electronic applications. Moreover, the employment of thermoplastic binder also reinforces the anti-folding ability, which renders the paper based electronic a better mechanical robustness. Considering the generality and low cost of the screen printing technique, our method seems more approaching to scalable production and practical uses.

**Table S2 A comparison of the recently reported electrical resistivity data and the current work**

|  | Paper substrate | Resistivity (Ω·cm) | Sheet resistant (Ω/sq) | Anti-folding/bending ability |
| --- | --- | --- | --- | --- |
| Screen printed ECA | Pulp paper | 6.2 ×10^-5^ | around 0.9 | More than 70 % conductivity can be preserved after 20 folding cycles |
| Writing conductive ink ^[1]^ | Printing paper | 2.0 ×10^-4^/4.3×10^-6^ (sintering) | --- | About 50 % conductivity can be preserved after 10 bending cycles (r=0.5 mm, not sintered) |
| Sputtering/Evaporating metal ^[2]^ | --- | 1.2×10^-5^ (tin) | around 0.1-10 | About 30 % conductivity can be preserved after 8 folding cycles |
| Desktop printed liquid metal ^[3]^ | Coated paper | 1.2×10^-5^ (gallium) | --- | More than 90 % conductivity can be preserved after 1 Bending cycles |
| Ink-jet printed conductive ink ^[4]^ | Nanostructure paper | 1.6 | --- | About 80 % conductivity can be preserved after 5000 bending cycles and 30 % conductivity can be preserved after 12 folding cycles |

Currently, we used surface sizing method to adjust the sizing degree of the paper (pulp paper). We found that with the increased amount of the added sizing agent (polyvinyl alcohol, PVA), the sizing degree changed dramatically. It seems difficult to control the sizing degree precisely. As the sizing degree increases dramatically, this variation become more significant. In this case, we can tell that at a low sizing level, the electrical conductivity of the printed ECA is positively correlated to the sizing degree. Instead, we found that the various commercial available paper kinds all seem to follows the modified Kirkpatrick model (formula 1), and their difference primarily lies in the f_loss_, as elucidated in the main text.


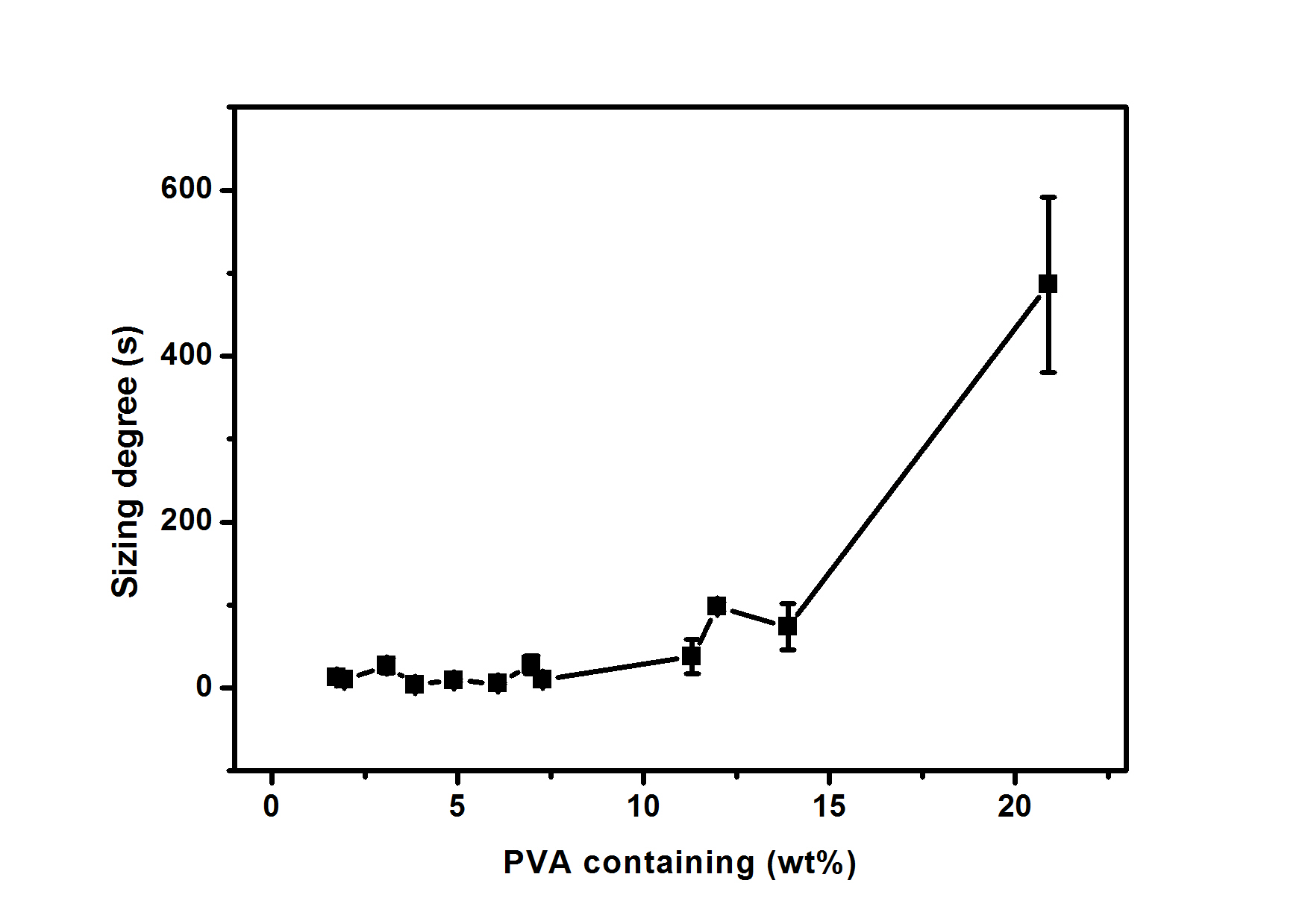


Figure S10 Relationship of sizing agent containing and sizing degree

References:

[1] A. Russo, B. Y. Ahn, J. J. Adams, E. B. Duoss, J. T. Bernhard and J. A. Lewis, *Advanced Materials*, 2011, 23, 3426-3430.

[2] A. C. Siegel, S. T. Phillips, M. D. Dickey, N. S. Lu, Z. G. Suo and G. M. Whitesides, *Advanced Functional Materials*, 2010, 20, 28-35.

[3] Y. Zheng, Z. Z. He, Y. X. Gao and J. Liu, *Scientific Reports*, 2013, 3, 1786.

[4] T. T. Nge, M. Nogi and K. Suganuma, *J. Mater. Chem. C*, 2013, 1, 5235-5243.

**Video S1 Foldable LED winking circuit on a printing paper substrate**

**Video S2 The LED winking circuit working in folded circumstance**
